# A Neuron-Based Kalman Filter with Nonlinear Autoregressive Model

**DOI:** 10.3390/s20010299

**Published:** 2020-01-05

**Authors:** Yu-ting Bai, Xiao-yi Wang, Xue-bo Jin, Zhi-yao Zhao, Bai-hai Zhang

**Affiliations:** 1 School of Computer and Information Engineering, Beijing Technology and Business University, Beijing 100048, China; baiyuting@btbu.edu.cn (Y.-t.B.); zhaozy@btbu.edu.cn (Z.-y.Z.); 2 Beijing Key Laboratory of Big Data Technology for Food Safety, Beijing Technology and Business University, Beijing 100048, China; 3 School of Automation, Beijing Institute of Technology, Beijing 100811, China; smczhang@bit.edu.cn

**Keywords:** kalman filter, nonlinear autoregressive, neural network, noise filtering

## Abstract

The control effect of various intelligent terminals is affected by the data sensing precision. The filtering method has been the typical soft computing method used to promote the sensing level. Due to the difficult recognition of the practical system and the empirical parameter estimation in the traditional Kalman filter, a neuron-based Kalman filter was proposed in the paper. Firstly, the framework of the improved Kalman filter was designed, in which the neuro units were introduced. Secondly, the functions of the neuro units were excavated with the nonlinear autoregressive model. The neuro units optimized the filtering process to reduce the effect of the unpractical system model and hypothetical parameters. Thirdly, the adaptive filtering algorithm was proposed based on the new Kalman filter. Finally, the filter was verified with the simulation signals and practical measurements. The results proved that the filter was effective in noise elimination within the soft computing solution.

## 1. Introduction

In the typical control systems, the measurement is the primary component that senses the system status and provides the input information. The measurement accuracy influences the control effect directly. Especially in intelligent terminals, such as industrial robots, unmanned aerial vehicles and unmanned vehicles, various sensors are implemented to measure the motion state and working condition. The sensors are expected to be of high precision. However, the precision is limited by the manufacturing technique when the intelligent terminals are with small size and low cost relying on the micro-electromechanical sensors [1,2]. For the complicated noises, it is essential to filter the noises. Then the motion state and condition can be estimated accurately for the target tracking and control.

In the noise filtering and state estimation field, many methods have been studied, such as the wavelet filter [3], time-frequency peak filtering [4], empirical mode decomposition [5] and the Kalman filter [6]. These filtering and estimation algorithms are often based on the mathematical models and established using the iterative schemes [7,8,9] or recursive schemes [10,11]. Some filtering-based estimation algorithms use input-output representations [12,13], and others use state-space models [14,15,16]. Among these methods, Kalman filter is a state estimation algorithm based on the state-space model. It introduces the state space into the stochastic estimation theory and obtains the optimal estimation without requiring a vast amount of historical data. However, it is obvious that the Kalman filter depends highly on some assumptions that the system model is linear, the process and measurement noises are standard Gaussian, and their covariance matrixes are all known. When these assumptions are seriously limited in reality, two categories of methods have been explored. On the one hand, the adaptive Kalman filter (AKF) was proposed focusing on the parameter adjustment to approximate the filtering process to the practical system. Then common AKF includes innovation-based adaptive estimation (IAE) [17], multiple model adaptive estimation (MMAE) [18] and adaptive fading Kalman filter (AFKF) [19]. On the other hand, some methods focus on nonlinear systems, such as extended Kalman filter (EKF) [20], unscented Kalman filter (UKF) [21], noise-robust filter [22] and other estimation methods [23,24]. The two categories of the methods try to describe and represent the system features with the variable approximation. The filters will be efficient if the alternative expression of the model and parameter is similar to the system dynamic characteristic.

The methods above mainly extract and represent the system characteristics with the existing information. While the characteristic extraction is the specialty of machine learning, the artificial neural network (ANN) has been introduced in noise filtering and state estimation. The leading solution is the distributed mode in which Kalman filter and ANN are applied separately in sequential order [25,26,27]. In the mode, the neural network mainly preprocesses or reprocesses the data before or after the filtering process. However, the inner relation in the Kalman filter has not been explored deeply with ANN. Then it becomes an issue on how to extract the relationship of parameters in the Kalman filter and optimize the filtering results with the limited existing information.

Because of the advantages in Kalman filter and the neural network, a new neuron-based Kalman filter is built in this paper. It mainly enhances the filtering process with the existing information. The potential numerical relation of the intermediate variables in the Kalman filter is explored with the feature extraction and nonlinear fitting ability of the neural network. In the paper, neurocomputing is integrated with the inner components of the Kalman filter. The nonlinear autoregressive model is introduced and constructed to predict and modify the critical intermediate variables in the Kalman filter. The simulation and practical experiments have verified the precision and feasibility of the proposed filter.

This paper is organized as follows: Section 2 introduces the underlying theory and related works on noise filtering. Section 3 presents the main proposed filter with the framework and network design. The simulation and experiment are designed and conducted in Section 4. The results and work are discussed in Section 5 and Section 6 finally concludes the paper.

## 2. Related Work

As the typical filtering method, the Kalman filter is selected as the basic framework in this paper. The basic theory and developments of the Kalman filter are introduced firstly. Then the related work is presented on the integration of the filter and neural networks.

### 2.1. Kalman Filter and Its Improvement

Because of its clearness and convenience in computer calculation, the Kalman filter has been the classical method in the filtering and estimation of Gaussian stochastic systems [28,29]. It is applied widely in target tracking [30], integrated navigation [31], communication signal processing [32], etc. Kalman filter introduces the state space description in the time domain, in which the estimated signal is set as the output of the stochastic linear system in the action of white noise. Kalman filter is appropriate for the stationary process and the non-stationary Markov sequence.

For the detailed analysis in the paper, the main algorithm of the Kalman filter is presented here. The discrete model can be expressed as:

(1) x(k+1)=A(k)x(k)+w(k)

(2) z(k)=C(k)x(k)+v(k)

where x(k) is the to-be-estimated variable or state variable, z(k) is the measurement value from sensors, A is the state transition matrix or process matrix, C is the measurement matrix, w(k) is the process noise, v(k) is the measurement noise. The concrete Kalman filter algorithm is shown as follows:

(1) State estimation updating:

(3) $$\hat{x}(k|k)=\hat{x}(k|k-1)+K(k)[z(k)-C(k)\hat{x}(k|k-1)]$$

(2) One step forward prediction:

(4) $$\hat{x}(k|k-1)=A(k-1)\hat{x}(k-1|k-1)$$

(3) Filtering gain calculation:

(5) $$K(k)=P(k|k-1)C^{T}(k)[C^{T}(k)P(k|k-1)C(k)+R(k)]$$

(4) The variance of the state estimation calculation:

(6) $$P(k|k-1)=A(k-1)P(k-1|k-1)A^{T}(k-1)+Q(k-1)$$

(7) P(k|k)=[I−K(k)C(k)]P(k|k−1)

where $\hat{x}(k|k)$ is the posterior estimation, $\hat{x}(k|k-1)$ is the prior estimation which is also called the prediction, K is the filtering gain, P is the variance of the state estimation, Q is the variance of the process noise, R is the variance of the measurement noise.

There are assumptions in Kalman filter, namely that the process and measurement noises are standard Gaussian noises, and their covariance matrixes are all known. The assumptions deviate from the real systems. Then many studies have been carried out to improve Kalman filter from different solutions.

Some improvements were proposed for the nonlinear system, and the typical methods include EKF [20] and UKF [21]. In EKF, the Taylor expansion of the nonlinear function is truncated with the first-order linearization, and other higher-order terms are ignored. Then the nonlinear problem can be transformed into the linearity, which is suitable for the Kalman filter. In UKF, the prediction measurement values are represented with the sampling points, and the unscented transformation is used to deal with the nonlinear transfer of mean and covariance. EKF and UKF have been improved, as well as the integration with other methods [33,34,35].

Aside from nonlinear system methods, AKF methods have been studied to solve problems where mainly the settled and experiential parameters are given. The representative IAE [17], MMAE [18], and AFKF [19] are proposed based on the thought that the model parameter and noise statistics are modified with the observation and judgment during the filtering process. From a literature search, it was seen that some improvements in AKF [36,37,38] were presented recently. In the latest work [38], the colored noise is analyzed with the adaptive parameter. The second-order adaptive statistical model and Yule-Walker algorithm are used to recognize and filter the noises. The work is one of the latest representative improvements of AKF, and it can be set as a contrast in the experimental research.

The two categories of methods above, nonlinear and adaptive filters, mainly improve the filtering performance from the approximate system modeling and parameter adjustment. They are conducted based on inherent mathematics and statistic derivation. They provide an effective solution to promote the Kalman filter in the system mechanism analysis idea. The filtering and prediction are based on the mathematical models by assuming that the model parameters are known or estimated using some parameter identification methods, including the iterative algorithms [39,40,41], the particle-based algorithms [42,43,44] and the recursive algorithms [45,46,47,48].

### 2.2. Filter with Neural Network

The methods in Section 2.1 improve the Kalman filter by modifying the system model and parameters based on the mathematic mechanism. The idea can be carried out with another data-driven solution. For the filtering parameter adjustment, the core task is to find and express the relation between parameters and process data, which meets the ability of neural networks. ANN has caused great concerns again with the trends of deep learning and artificial intelligence. ANN can fit the nonlinear model with excellent performance. It can solve the nonlinear and time-varying problems without a concrete internal mechanism model. For the problematic modeling of process and noise in the Kalman filter, ANN can be considered as a helpful tool to reconstitute the unknown elements in the filter. Scholars have made some efforts to explore the integrations of ANN and Kalman filter. The related research can be divided into two categories, including the distributed and crossed integration.

#### 2.2.1. Distributed Integration of Kalman filter and ANN

For the distributed integration, Kalman filter and ANN are applied separately in sequential order. Liu et al. [25] smoothed the measurement value with the Kalman filter, and the filtered results were set as the input of the backpropagation neural network (BPNN). Hu et al. [49] estimated the target location with Kalman filter and the estimation was imported into BPNN to classify the targets. Liu et al. [50] utilized Kalman filter and fuzzy neural network (FNN) in a multi-source data fusion framework of an adaptive control system, in which data was processed firstly with Kalman filter, and the filtered results were set as the input of FNN. Others [26,27,51] used a Kalman filter and ANN in reverse order, in which ANN is constructed before the Kalman filter. Leandro et al. [26,27] built up BPNN to predict a variable, which is an important state variable in the Kalman filter. Cui et al. [51] proposed a radial basis function neural network (RBF) to train the GPS signals, and the RBF output is the input of adaptive Kalman filter, aiming at improving the processing precision.

#### 2.2.2. Crossed Integration of Kalman Filter and ANN

Different from the methods above, Kalman filter and ANN are combined in a tight pattern, in which ANN is applied during the internal filtering procedure. Shang et al. [52] predicted the model error in the filter with FNN, and the error level was considered to confirm the measurement noise covariance, which was set as 0 or infinity. Li et al. [53] thought that the gain of EKF was usually modified with the erroneous measurement, which reduced the gain precision. They used BPNN to train the gain with the input of measurement, estimation, and error, and then the precision was increased. Deep neural networks [54] have been studied recently. Pei et al. [55] combined a deep neural network with the Kalman filter in the emotion recognition of the image and audio. The features extracted by the deep neural network were input into the switching Kalman filter to obtain the final estimation results.

In the research reported in the literature, more works are conducted in the first separately distributed mode, in which Kalman filter and ANN process the data respectively. The mode does not adjust the inner parameters of the Kalman filter. The works of tightly crossed integration are relatively few. They can be improved with the relation exploration in filter parameters with ANN. Besides, the category and structure of the neural network can be studied to meet the demand for filtering calculation procedures.

## 3. Neuron-Based Kalman Filter

### 3.1. Framework of Neuron-Based Kalman Filter

Kalman filter provides a feasible framework to filter the noises and estimate the system state. The components of the Kalman filter can be divided into two categories, namely the models and intermediate variables. The models describe the system dynamic and the measurement process, including the system process equation and measurement equation. The intermediate variables influence the filtering results seriously, but they are difficult to determine in practice. In this paper, the effect of the intermediate variables on the filtering is explored with neural networks. The neurons are integrated into the Kalman filter, and the neurons can help to optimize the filtering process with the limited existing information.

**Figure 1:**
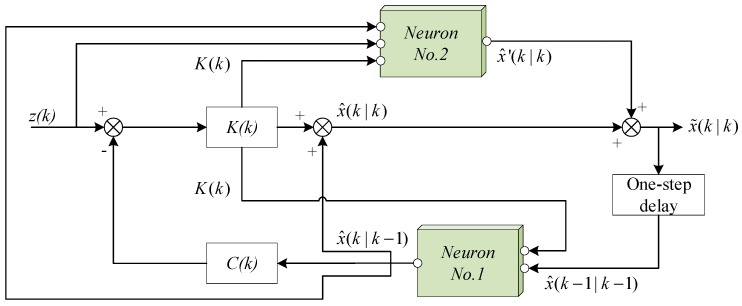
Framework structure of the neuron-based Kalman filter.

The main influence factors of Kalman filter include the process equation, process noise, and measurement noise, expressed as the matrix A, Q and R. Notably, the noise variances are the critical intermediate variables that affect the estimation results. The effect of noise variances is expressed in the filtering gain K, and the filtering gain determines the estimation result as an important weight. In the optimizing thought with the neurons, the influence relation of the filtering results and the variables should be explored. Then the framework of the integrated Kalman filter is designed firstly, shown in Figure 1, and the concrete design ideas will be interpreted later.

Considering the prediction process of the Kalman filter in Equation (4), the estimation result is affected by the precision of the process equation. The process equation describes the system change along the time, but the common simplified equation is difficult to model the actual system. In the view of data-driven, the core of the process equation is the time series relation of the system change. And the time series can be modeled well with neural computing. Then a neuro unit is introduced to model the process in a black-box thought. The first neuro unit can be expressed as:

(8) $$\hat{x}(k)=f_{1}(K(k),K(k-1),K(k-2),\cdots ,\hat{x}(k-1),\hat{x}(k-2),\cdots )$$

The inputs in the first neuro unit are the filtering gain K series and the state estimation value $\hat{x}$ at the previous time points, represented with k−1,k−2,⋯. The output is the estimation $\hat{x}$ at the time k. The key to the model is the fitting function $f_{1}$ which aims at the excavation of the time series features in the estimated variables and intermediate filtering variables. As a vital variable, the filtering gain K is obtained from the variance of process and measurement noise Q and R, and it can represent the noise features to some extent. Therefore, the filtering gain is set as an input to transmit the noise features to the system process model. The output estimation value is related to the state at the previous time and the filtering gain. Then the new estimation can be regarded as a more accurate predictive value of the system, and it replaces the initial prediction value in Equation (4) to continue the computing process of the Kalman filter.

For neural computing, it needs training with the existing data. In the training of the first neuro unit, the filtering process data and the final estimation result are collected as the training set. In detail, the series data of the filtering gain from time step (k−m) to k and the estimation value from (k−m) to (k−1) are set as the training input data, where m is the prediction length set in the neural computing. The estimation value at k is set as the training output. In fact, the previous one-step prediction value is optimized with the final estimation value in the network. The fitting function $f_{1}$ can be obtained with the training data and the learning algorithm which will be discussed in Section 3.2.

Considering the final estimation process of Kalman filter in Equation (3), the estimation result is determined by two parts, of which one is the prediction value, and the other one is the measurement residual error. The second neuro unit is built to discover the mapping relation between the two parts and the final estimation result, expressed as:

(9) $${\hat{x}}^{\prime }(k)=f_{2}(K(k),K(k-1),\cdots ,z(k),z(k-1),\cdots ,\hat{x}(k),\hat{x}(k-1),\cdots )$$

The inputs of the second neuro unit are the measurement value z, the prediction value $\hat{x}$ and the filtering gain K at the previous time points, represented with k−1,k−2,⋯ The output is the final estimation value ${\hat{x}}^{\prime }$ at the time k. The final estimation synthesizes the measurement and the filtering intermediate variables such as the noise variance matrix (reflected by the filtering gain) and the prediction variables. The function *f*_2_ is trained to realize the synthetization. In the training, the values of K, z, $\hat{x}$ from (k−m) to k are set as the input, and the final estimation value ${\hat{x}}^{\prime }$ at k is set as the output.

It can considered that the estimation via the neuro units is an effective supplement and amendment of the estimation in Kalman filter. Then the new final estimation value can be obtained by synthesizing the two estimation values from the neural computing and Kalman filter:

(10) $$\hat{x}(k|k)=(1-\alpha )\hat{x}(k|k)+\alpha {\hat{x}}^{\prime }(k|k)$$

where $\hat{x}(k|k)$ is from Kalman filter, ${\hat{x}}^{\prime }(k|k)$ is from the neuro unit. (1−α) and α are the weights of the two estimation values. α is determined by the validation error of the neuro unit, and:

(11) $$\alpha =\frac{n}{\sum_{i=1}^{n}\left|\left(d_{i}^{v}-d_{i}\right)/d_{i}\right|}$$

where d is the validation set during the neuro unit training, $d^{v}$ is the output of the neuro unit for the validation set, n is the number of data in the validation set.

### 3.2. Neuro Units Based on Nonlinear Autoregressive Model

In the framework of the neuron-based Kalman filter, the critical components are the neuro units which analyze the intermediate variables to support the final filtering result. Referring to the demand analysis of the two units, the two functions in Equations (8) and (9) should be able to fit the nonlinear relation in multiple variables. Moreover, they should excavate the time-series features in the data. With the two aspects of the demands, the nonlinear autoregressive model with exogenous input (NARX) can be the appropriate solution [56,57]. NARX derives from the time series autoregressive analysis, and it is effective in the reconstitution of the nonlinear systems. The availability of NARX has been proved by various applications [58,59,60].

**Figure 2:**
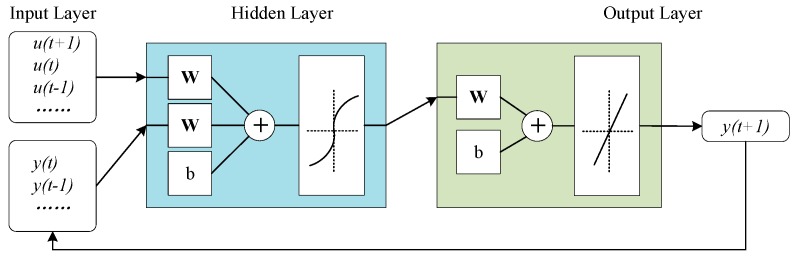
Structure of NARX.

NARX belongs to the recurrent neural network. It has a learning efficiency with the better gradient descent. The nonlinear relation between the inputs and outputs in NARX can be expressed as follows:

(12) y(t+1)=ϕ(y(t),y(t−1),⋯,y(t−n),u(t+1),u(t),⋯,u(t−m))

where y(t+1) is the output to be predicted, y(t) to y(t−n) are the historical outputs, u(t+1) to u(t−m) are the related inputs which last to the current moment. ϕ represents the nonlinear relation between inputs and outputs, and it also represents the structure of NARX, shown in Figure 2.

NARX consists of the input layer, hidden layer, and output layer. The transfer function of NARX is similar to the backpropagation neural network, and a one-hidden-layer network is shown as follows:

(13) $$f(\cdot )=g(\sum \omega_{h}h(\cdot ))$$

(14) $$h(\cdot )=r(\sum \omega_{i}u_{i})$$

where g and r are the activation functions of the output, $\omega_{h}$ is the node weight in the hidden layer, h(⋅) is the activation function of the hidden layer, $\omega_{i}$ is the weight of all inputs. The vector form of $\omega_{h}$ and $\omega_{i}$ are represented with *W* in Figure 2.

**Figure 3:**
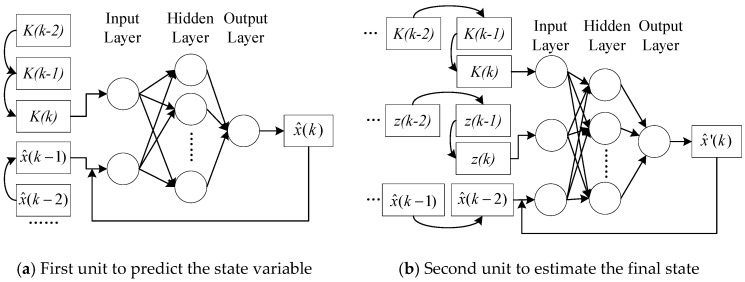
Concrete structures of the two neuro units in the proposed Kalman filter.

Based on the primary NARX structure, the two neuro units in the neuron-based Kalman filter can be designed concretely, which are shown in Figure 3.

For the neuron units in Figure 3, the concrete scale can be determined with the traditional empirical mode in the shallow neural network. The number of the hidden layer is set as one according to the number of input and output variables. The number of hidden nodes can be determined with the equations, such as $n=\log_{2}p$, $n=\sqrt{p+q}+a$, where n is the number of hidden nodes, p is the number of input nodes, q is the number of output nodes, and a is a constant between 1 and 10. Besides, the number of hidden nodes can be adjusted in the network training, following the network performance.

Based on the static construction of the neural network, the appropriate training method should be selected to obtain favorable dynamic performance. The gradient descent method is the core solution in neural network training. Some improved algorithms have been proposed. Levenberg- Marquardt (L–M) [61,62] is a rapid training method that combines the basic gradient descent method and Gauss-Newton method. Its error target function is:

(15) $$E(w)=\frac{1}{2}\sum_{i=1}^{p}{\left\|Y_{i}-Y_{i}{}^{\prime }\right\|}^{2}=\frac{1}{2}\sum_{i=1}^{p}e_{i}^{2}(w)$$

where $Y_{i}$ is the expected output, $Y_{i}{}^{\prime }$ is the actual output, $e_{i}(w)$ is the error, p is the number of samples, w is the vector consisting of network weights and threshold values. 

The *k*-th iterative vector of weights and threshold values is $w^{k}$, and the new vector is:

(16) $$w^{k+1}=w^{k}+\Delta w$$

and the increment in L–M is calculated as:

(17) $$\Delta w={\left[J^{T}(w)J(w)+\mu I\right]}^{-1}J^{T}(w)e(w)$$

where I is the unit matrix, μ is the learning rate, J(w) is the Jacobian matrix, and:

(18) $$J(w)=\left[\begin{array}{cccc} \frac{\partial e_{1}(w)}{\partial w_{1}} & \frac{\partial e_{1}(w)}{\partial w_{1}} & \cdots & \frac{\partial e_{1}(w)}{\partial w_{1}}\\ \frac{\partial e_{1}(w)}{\partial w_{1}} & \frac{\partial e_{1}(w)}{\partial w_{1}} & \cdots & \frac{\partial e_{1}(w)}{\partial w_{1}}\\ \vdots & \vdots & \ddots & \vdots \\ \frac{\partial e_{1}(w)}{\partial w_{1}} & \frac{\partial e_{1}(w)}{\partial w_{1}} & \cdots & \frac{\partial e_{1}(w)}{\partial w_{1}} \end{array}\right]$$

With the training method, the neuro units can be built with the anticipative functions. Then the improved Kalman filter can be established with the functional neuro units.

### 3.3. Adaptive Filtering Algorithm

**Figure 4:**
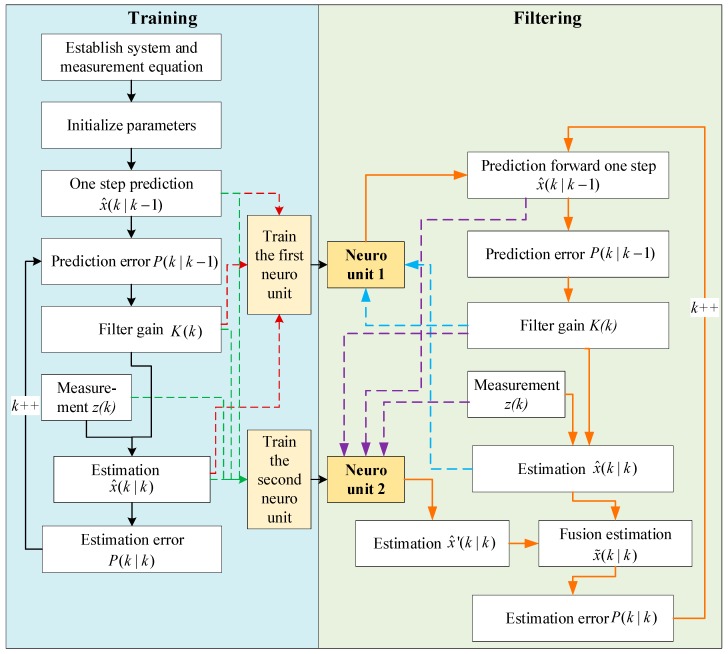
Algorithm flow of the adaptive filtering with the neuron-based Kalman filter.

In the framework of neuron-based Kalman filter, two neuron units are introduced into the basic consistent of Kalman filter. The input, output, and inner structure of the neuro unit are designed to improve the filtering. Finally, the adaptive filtering algorithm based on the improved Kalman filter is proposed here, in which the neuro units are trained firstly and applied to the filter. The flow of the algorithm is presented in Figure 4.

As shown in Figure 4, the algorithm consists of two parts, namely the training process on the left and the filtering process on the right. The concreter flow of the algorithm is as follows:

(A) Training process
  (1) The system and measurement equations are established according to the object. The parameters in the Kalman filter can be initialized with empirical values.
  (2) The primary calculation of the Kalman filter is conducted iteratively following Equations (1)–(7). The measurement vectors are imported into the filter along with time. The intermediate and final values are recorded, including the one-step prediction value, Kalman gain, measurement, estimation result, etc. The recorded values are all labeled with a time stamp. Meanwhile, the iterative steps should be no less than about 150 for the following neuro unit training. The number of sample steps may be adjusted according to the complexity of signals.
  (3) With the filtering values in step 2, they are marked with the step number to form the time series sets. Then the prediction value and filter gain are imported into the first neuro unit. The prediction value, filter gain, and measurement are imported into the second neuro unit. The estimation result is set as the reference output of the two units.
  (4) The neuro units are trained with the learning method L–M in Section 3.2. The trained neuro units are obtained when the preset iteration conditions are met, including the numbers of iteration or the convergence error.
(B) Filtering process with trained neuro units
  (5) Based on the model equations and the initialized parameters in Kalman filter, the initial variable and filter gain are imported into the first neuro unit, and the prediction value is outputted and set as the basis of prediction error.
  (6) The filter gain is updated and used to calculate the estimation value with the measurement. Meanwhile, the prediction value, filter gain, and measurement are imported into the second neuro unit to obtain another estimation value.
  (7) The two estimation values are fused following Equation (10).
  (8) The step moves forward to conduct steps (5)–(7) iteratively. In the iteration, the measurement vectors are calculated along with time.

## 4. Experiment and Result

The simulation and practice experiments are conducted to test the filter proposed. In the simulation, different noises are generated to simulate the complex noises in the sensors. In the experiment, the wheeled robot path is measured with low-cost GPS. All the computing runs with MATLAB 2017a on a PC equipped with an Intel Core i5-6200U CPU@2.30 GHz and 8 GB RAM. The experiment setting and results are presented in this section.

### 4.1. Simulation and Result

The common noises in sensors are the white noise and color noise. The signals with the two kinds of noises are generated in the simulation. The two sets of the signals are:

(19) $$x_{1}(k)=\sigma (k)f(k)$$

(20) $$x_{2}(k)=G(z^{-1})f(k)$$

where f(k) is the Gaussian white noise. σ(k) is the standard deviation of f(k), and σ(k)=(L+k)/L, L is the number of signal samples, and k is the sample number. $G(z^{-1})$ is the transfer function of a system which can be second order or third order to simulate the noise change process.

**Figure 5:**
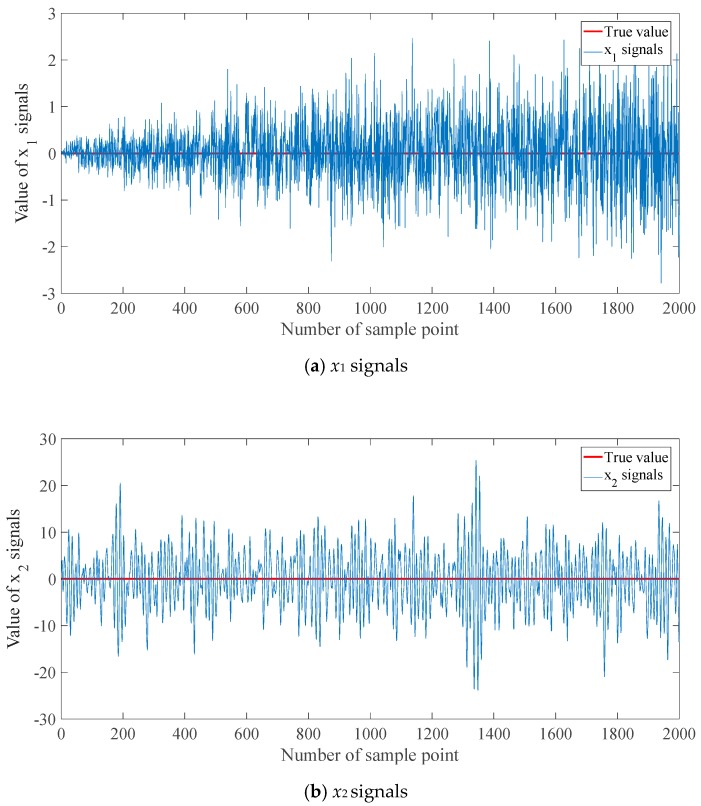
Simulation signals with different noises.

The first set is the approximately linear noises, and the second one is the sinusoidal noises, corresponding to the white noise and color noise, respectively. In the simulation, the sampling interval is 0.02 s. The numbers of signal samples are all 2000. That is, the sampling time is the 40 s. The simulation signals are shown in Figure 5, in which the true values of *x*_1_ and *x*_2_ are 0.

In the filtering of the simulation signals, the system model is established with the classical Jerk model, which also can be replaced with other motion models such as constant velocity, constant acceleration, Singer, interacting multiple model algorithms, and so on. For the Kalman filter, the initial state estimate *x*_0_ and covariance *P*_0_ are assumed to be *x*_0_ = [0 0 0]*^T^* and *P*_0_ = 1000*eye(4).

**Figure 6:**
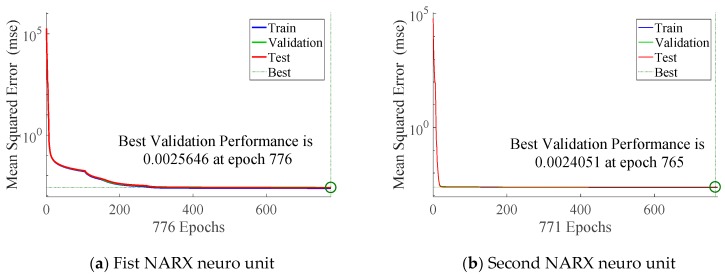
Training results of NARX in integrated filter.

Because the neuro unit needs the training, the first 70% of the data are set as the training data, and the rest is used to test the filtering result. In the setting of the two neuro units, the number of hidden nodes are set as 3 and 6, respectively. Other settings are also tested to obtain the optimal performance. The training results of NARX are shown in Figure 6.

**Figure 7:**
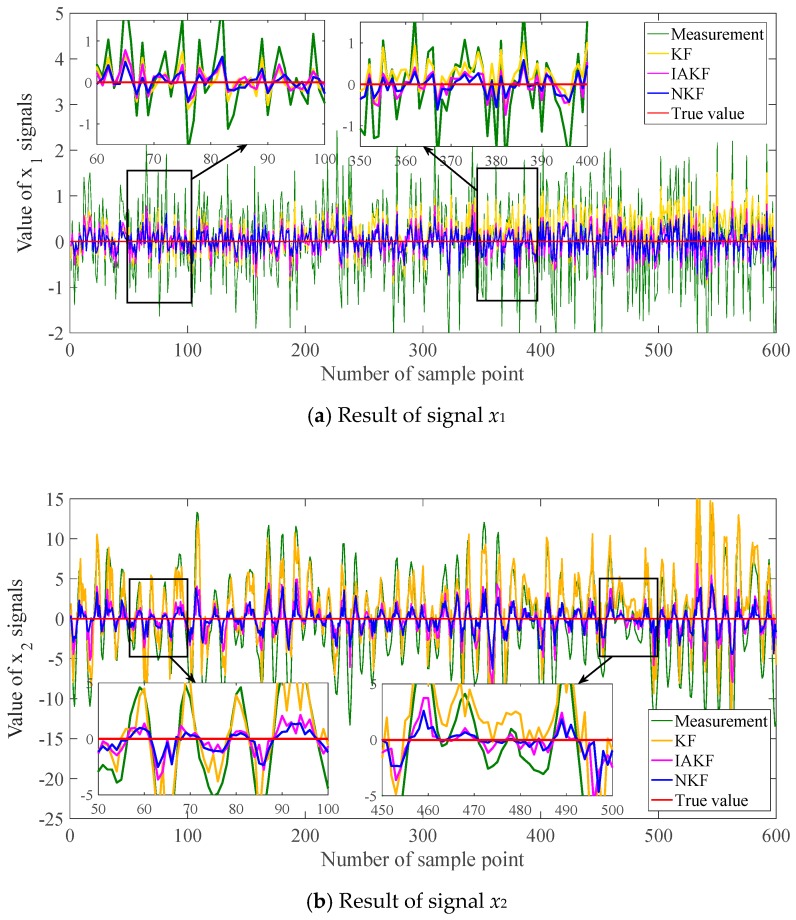
Filtering results of simulation signals.

Based on the trained neuro units, the data are imported into the proposed filter to estimate the variable values. For verifying the estimation performance, the traditional Kalman filter is set as a contrast, abbreviated as KF. Moreover, the proposed filter can be regarded as a solution to adaptive filtering. Then one of the latest improvements of AKF in [38] is also set as the contrast, abbreviated as IAKF. The proposed filter in this paper is abbreviated as NKF. The filtering results are shown in Figure 7. For the quantitative evaluation of errors, the mean absolute error (MAE) and root-mean-square error (RMSE) are calculated and listed in Table 1.

For the first set of signals which are of the white noise, the results of the three methods are relatively similar in the curve graph. NKF performs better slightly than the others. The trend is evident in the error evaluation criteria. MAE represents the mean level of errors. MAE of NKF declines 45.72% of KF and 21.41% of IAKF. RMSE shows the fluctuation degree of errors. RMSE of NKF decreases 45.23% of KF and 20.91% of IAKF.

For the second set, the filtering results show the distinguishable trends. The results of KF fluctuate sharply and become diverging in the latter period. IAKF and NKF can trace the signals more closely, and NKF is more effective in the intuitionistic graph. MAE of NKF has been reduced by 69.16% of KF, and 18.80% of IAKF. The decreasing percentage of RMSE reaches 67.77% and 21.13% for NKF to KF and IAKF. The error reduction of the second set is larger than the first set.

### 4.2. Practical Experiment and Result

**Figure 8:**
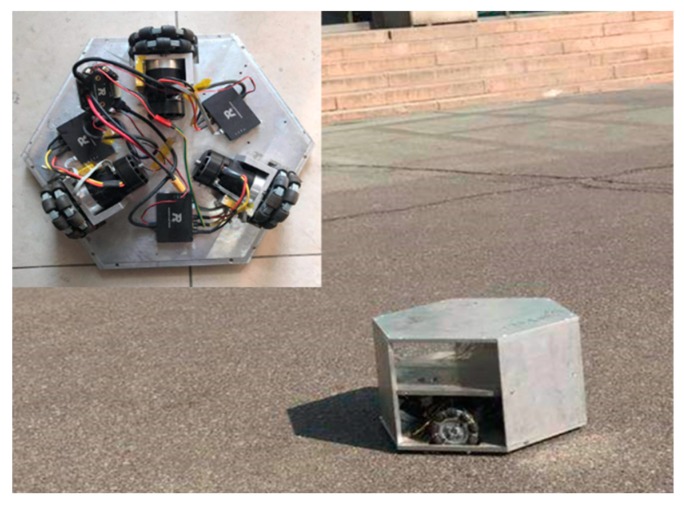
Wheeled robot used to measure the trajectory, and the robot is developed and assembled by laboratory of system engineering in Beijing Institute of Technology, Beijing, China.

**Figure 9:**
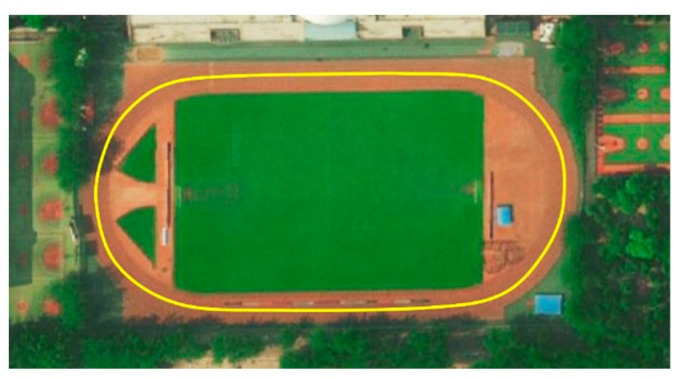
Presupposed trajectory in the practical experiment.

**Figure 10:**
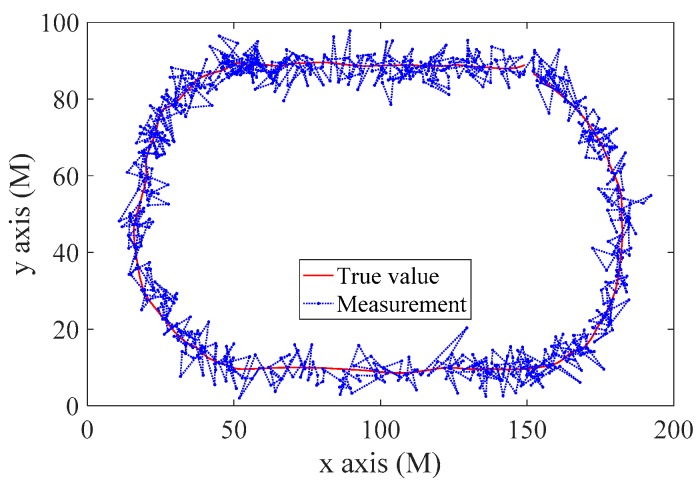
Relative coordinates transformed from the practical trajectory measurements.

Except for the simulation, a practical experiment is also conducted to verify the proposed method. A trajectory of the wheeled robot (shown in Figure 8) is measured on the playground, and the presupposed trajectory is presented in Figure 9. The robot started from the top right corner and ended at the same point. A low-cost GPS receiver is used to obtain the location information, including the longitude and latitude. The relative coordinates are transformed from the longitude and latitude:

(21) $$d=111.12\cdot \cos\frac{1}{\sin\phi_{t-1}\sin\phi_{t}+\cos\phi_{t-1}\cos\phi_{t}(\lambda_{t}-\lambda_{t-1})}$$

where d is the displacement, ϕ is the latitude and λ is the longitude. The displacement can be decomposed into the coordinates on a plane. The measurements and true trajectory in the relative coordinates are shown in Figure 10.

In this part, the data of the whole trajectory is filtered firstly. Then a segment of the trajectory in another measurement is tested again.

#### 4.2.1. Result of the Whole Trajectory

**Figure 11:**
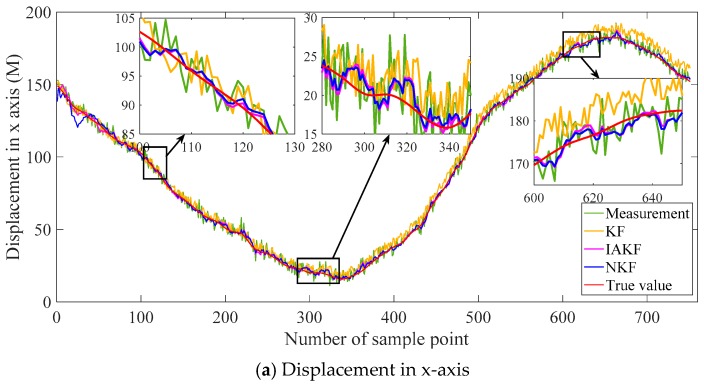

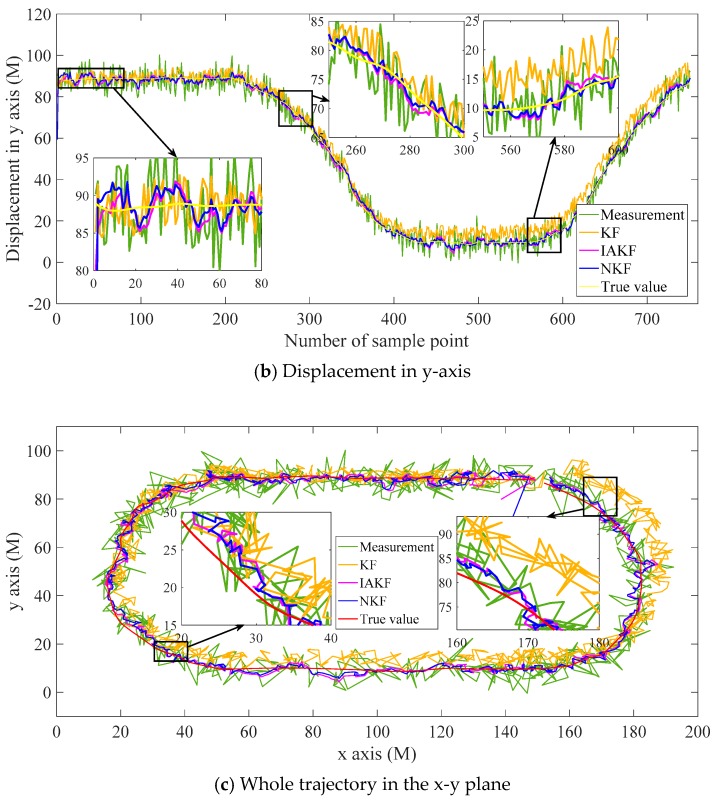
Filtering results of the whole trajectory.

**Figure 12:**
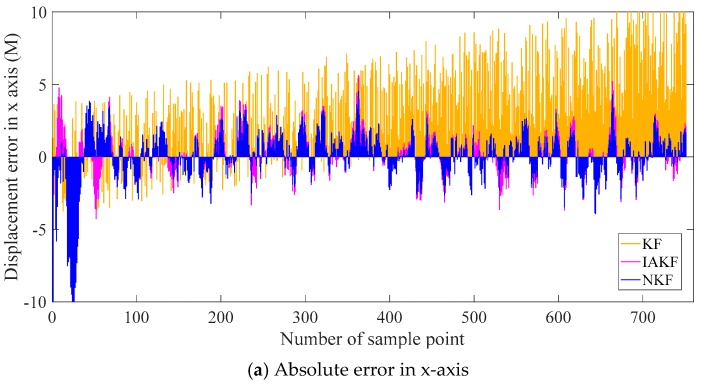

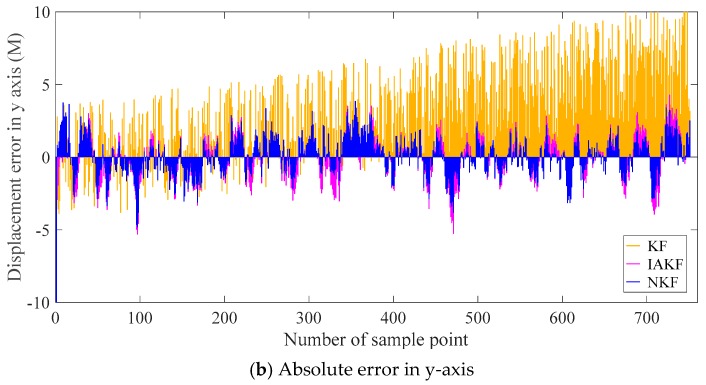
Absolute errors of displacement in x and y axes.

Similar to the simulation, the traditional Kalman filter and improvement of AKF in [38] are set as the contrast methods. The filtering results are shown in Figure 11, including the distance in the x-axis, y-axis, and x-y plane. The absolute errors are shown in Figure 12, and the evaluation indexes are in Table 2.

The results in Figure 11 show intuitively the small differences in the filtering results, and it is because the difference is in the order of magnitudes less than 1 m. The differences are more evident in the absolute errors in Figure 12. The general trends of the filtering results with the three methods are similar to the pattern of the second set signal in the simulation. It is because that the noises in the real sensors mainly are the color noises instead of the white noises. The basic Kalman filter performs badly in the practical system. Its results are unsteady and diverging along with the time. NKF perform better than others in various time periods besides the beginning. The neuro unit based on NARX reaches stable performance after the drastic fluctuation, which usually occurs at the beginning. Therefore, NKF is superior to the Kalman filter on the whole without the initial period. The performance can be analyzed quantitatively with the error criteria in Table 2.

The general trend is consistent in the *x*-axis and *y*-axis for the three indexes. There is a conspicuous promotion in MAE for NKF and the RMSE of NKF declines about 6% with the Kalman filter and 8% with IAKF.

#### 4.2.2. Result of Segment in the Trajectory

To test the proposed filter with more data, a segment of the whole trajectory is selected from one of the multiple measurements, which is not in the same measurement with the whole trajectory above.

**Figure 13:**
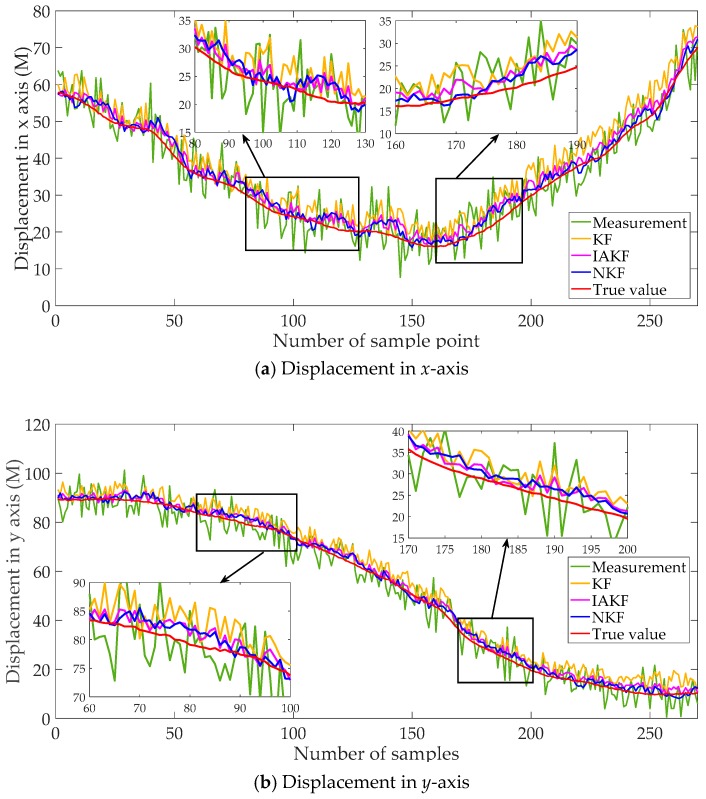
Filtering results of the selected segment of the trajectory.

The data from 180 s to 450 s are selected, including the displacements in x-axis and y-axis. The contrast methods are the same as the experiment above. The filtering results are shown in Figure 13, and the evaluation indexes of errors are listed in Table 3.

It can be found from Figure 13 that the general trend is similar to the results of the whole trajectory in Figure 11. The results of NKF and IAKF are intuitively approximately the same. From careful and detailed identification in the magnifying subfigure, the proposed NKF can filter the noises closer to the true value than other methods. In the results of the x-axis and y-axis, the basic KF performs badly with the obvious deviation.

The filtering performance can be evaluated more accurately with Table 3. For results in the x-axis, MAE of NKF is 32.73% of KF, 82.01% of IAKF. RMSE of NKF is similar to IAKF, but relatively lower than KF. The trend of results in the y-axis is consistent with the ones in x-axis. Compared with the error indicators in Table 2, the errors of the segment are larger than the whole trajectory, which may be due to the fewer data to train the neural networks.

## 5. Discussion

In this paper, a novel Kalman filter is designed by introducing neural computing. Simulations and experiments are carried out, and the results are presented and described briefly. Following the results, the methods are discussed in this section.

From the filtering results of simulation signals and practical measurements, it can be proved that the proposed filter can eliminate the noises to the anticipated degree. It performs distinctly better than the traditional Kalman filter, especially for complex noises. Besides, the proposed filter can achieve the latest improvements of AKF. The core thought of the proposed filter is to obtain more knowledge from the existing limited data during the filtering procedure. The process variables in the filtering are reutilized with the neural units, while the reutilization in AKF [36,37,38] is conducted with statistical methods. Therefore, the proposed filter can be regarded as a new exploration of parameter adjusting, which is similar to the essential thought of AKF.

In the proposed filter, the neuro unit is built based on the nonlinear autoregressive model. The neuro unit specializes in the nonlinear time-series feature extraction with a small-scale structure. Although many more networks have been proposed, it should be conservative in the selection of networks. The complex network may destroy the efficient and straightforward features of the Kalman filter. Besides, the complex network may be not suitable for the terminal applications without the high-performance processor.

Except for the intuitive estimation results, the computational complexity can be analyzed for the proposed and contrast method. According to the basic evaluation method of computational complexity, the complexity of KF, IAKF, and NKF is *O*(*n*^2^), *O*(*n*^2^) and *O*(*n*^3^), respectively, where *n* is the number of state variables. The complexity of NKF increases because dual matrix multiplication is introduced by neuro units. The operation time is also recorded in the experiment, shown in Table 4.

For computing time, the methods distinct slightly, although the complexity of NKF is higher. However, an important fact that cannot be ignored is the training of the neuro units. The time above is the test procedure, while NKF needs prior training. The training time is between 3 s and 7 s, according to the preset convergence conditions. In this paper, the training requires historical data in the offline mode. The filtering is conducted after the training, which reduces the real-time performance. It is the challenge how to realize online learning along with the filtering process, which can be studied in the future.

The neurons in the proposed method work well from the experiment results. Although a good filtering performance has been obtained, the inherent mechanism of the proposed method is actually not completely clear. Hence the theoretical analysis should be conducted, and the effect of the neural network on the filter should be deduced in the view of numerical analysis in the future.

In the proposed method, ANN inspired us to optimize the intermediate factors and calculating process in the Kalman filter with the black-box thought. It mainly solves the problem of modeling and parameter adjusting of the traditional filter. It can be a useful tool in the target tracking, trajectory estimation, and pedestrian navigation, especially in the situations of inexperienced modeling of complex systems and the parameter settings.

## 6. Conclusions

For the intelligent terminals and objects in the internet of things, it has been the vital task to sense the environment and self-status accurately. An improved Kalman filter is proposed with neural computing for accurate sensing. Kalman filter provides a favorable framework in which the system model can be replaced according to the concrete applications. The neuro unit based on NARX is a powerful tool to examine nonlinear and time-series relations. The proposed filter focuses on the data change features and tries to lower the impact of model analysis. In future work, the stability and rapidity of neural computing should be studied deeply. The neuron-based Kalman filter can develop more fully with smarter and faster online neural computing. Moreover, the theoretical derivation should be carried out to support the neuro-based filter. The proposed method can combine other identification approaches [63,64,65,66] to study the modeling and filtering problems of other dynamic time series and stochastic systems with colored noises [67,68,69,70], and can be applied to other fields [71,72,73,74], such as signal modeling and control systems [75,76,77,78,79] studied in other literature [80].

## Figures and Tables

**Table 1:** Evaluation of filtering errors.

		KF	IAKF	NKF
Signal *x*_1_	MAE	0.3692	0.2550	0.2004
	RMSE	0.4577	0.3170	0.2507
Signal *x*_2_	MAE	3.3379	1.2678	1.0294
	RMSE	4.4763	1.8295	1.4429

**Table 2:** Evaluation of filtering errors (whole trajectory).

		KF	IAKF	NKF
x axis	MAE	3.8730	1.3117	1.3048
	RMSE	4.6732	1.9079	1.6594
y axis	MAE	3.7327	1.3184	1.1651
	RMSE	4.5560	1.7578	1.6430

**Table 3:** Evaluation of filtering errors (segment of the trajectory).

		KF	IAKF	NKF
*x* axis	MAE	4.0157	2.0905	1.7159
	RMSE	4.7610	2.5017	2.0879
*y* axis	MAE	3.8769	1.7897	1.5230
	RMSE	4.5707	2.1330	1.8024

**Table 4:** Operation time of different methods in simulation and experiment (time unit: s).

	Simulation		Practical Experiment (Whole Trajectory)	
	Signal *x*_1_	Signal *x*_2_	*x* Axis	*y* Axis
KF	1.23	1.45	2.15	2.09
IAKF	1.37	1.73	2.32	2.43
NKF	1.27	1.79	2.41	2.24
